# Distinct adaptor proteins assist exit of Kre2-family proteins from the yeast ER

**DOI:** 10.1242/bio.20146312

**Published:** 2014-02-28

**Authors:** Yoichi Noda, Takehiro Hara, Minako Ishii, Koji Yoda

**Affiliations:** Department of Biotechnology, University of Tokyo, Yayoi, Bunkyo-Ku, Tokyo 113-8657, Japan

**Keywords:** Golgi, Endoplasmic reticulum, COPII vesicle proteins, Erv41, Erv46, Ktr4, Svp26

## Abstract

The Svp26 protein of *S. cerevisiae* is an ER- and Golgi-localized integral membrane protein with 4 potential membrane-spanning domains. It functions as an adaptor protein that facilitates the ER exit of Ktr3, a mannosyltransferase required for biosynthesis of *O*-linked oligosaccharides, and the ER exit of Mnn2 and Mnn5, mannosyltransferases, which participate in the biosynthesis of *N*-linked oligosaccharides. Ktr3 belongs to the Kre2 family, which consists of 9 members of type-II membrane proteins sharing sequence similarities. In this report, we examined all Kre2 family members and found that the Golgi localizations of two others, Kre2 and Ktr1, were dependent on Svp26 by immunofluorescence microscopy and cell fractionations in sucrose density gradients. We show that Svp26 functions in facilitating the ER exit of Kre2 and Ktr1 by an in vitro COPII budding assay. Golgi localization of Ktr4 was not dependent on Svp26. Screening null mutants of the genes encoding abundant COPII membrane proteins for those showing mislocalization of Ktr4 in the ER revealed that Erv41 and Erv46 are required for the correct Golgi localization of Ktr4. We provide biochemical evidence that the Erv41-Erv46 complex functions as an adaptor protein for ER exit of Ktr4. This is the first demonstration of the molecular function of this evolutionally conserved protein complex. The domain switching experiments show that the lumenal domain of Ktr4 is responsible for recognition by the Erv41-Erv46 complex. Thus, ER exit of Kre2-family proteins is dependent on distinct adaptor proteins and our results provide new insights into the traffic of Kre2-family mannosyltransferases.

## INTRODUCTION

Most of the newly synthesized membrane and secretory proteins are either inserted in the ER or translocated across the membrane into the lumen of the ER, and leave for the *cis*-Golgi by COPII vesicles (Antonny and Schekman, 2001; Barlowe, 2002; Wickner and Schekman, 2005). To efficiently export proteins that function in organelles other than the ER or outside of the cell from the ER is very important for optimal cell growth. It has been reported that some membrane proteins possess motifs that are recognized by one of the COPII coat components, Sec24, and these proteins are incorporated efficiently into COPII vesicles via the direct interactions with the coat during the budding process (Miller et al., 2003; Mossessova et al., 2003). Other membrane proteins with no such motifs or soluble secretory proteins are recognized and captured in the ER by membrane proteins called cargo receptors or adaptors (Dancourt and Barlowe, 2010; Noda and Yoda, 2013). The cargo adaptor proteins are typically defined by an ability to bind both coat components and cargo proteins, and the simultaneous interaction facilitates the cargo loading and ER exit by the COPII vesicles. Analysis of membrane proteins enriched in the COPII vesicles, which are named Emp or Erv proteins, identified several adaptor proteins and cargo proteins, with the latter depending on the former for an efficient ER exit (Otte et al., 2001; Muñiz et al., 2000; Sato and Nakano, 2002). A recent large-scale study using an automated microscopic system further revealed a novel adaptor and cargo pairs, and found that, in particular, Erv14 had many cargo proteins compared to other adaptor proteins identified so far and thus appeared to play more important roles in cargo export from the ER (Herzig et al., 2012). Despite these studies, whether adaptor proteins identified to date can facilitate ER export of all proteins destined for various intracellular locations, how the interactions between adaptor and cargo proteins are regulated, or whether the universal sequence motifs located in the adaptor or cargo proteins are used for their associations, remains mostly unknown.

Svp26 is a yeast ER-Golgi membrane protein that we discovered through a proteomics analysis of Golgi membrane proteins (Inadome et al., 2005) and the homologous proteins are conserved across species. Deletion of the *SVP26* gene does not affect yeast growth, but leads to the hyperglycosylation of *N*-linked glycans. Also, in Δ*svp26* cells, the distribution of mannosyltransferases, Ktr3, Mnn2 and Mnn5, changes significantly from the Golgi to the ER patterns (Inadome et al., 2005; Noda and Yoda, 2010). Biochemical analyses revealed that Svp26 functions as an adaptor protein to assist the ER exit of these proteins (Noda and Yoda, 2010; Bue and Barlowe, 2009). Other laboratories also reported that Svp26 facilitates ER exit of the membrane proteins, Pho8 (Bue et al., 2006) and Gda1 (Anand et al., 2009). Interestingly, all of the Svp26-dependent cargo proteins identified to date are type II membrane proteins.

Ktr3 is a member of the Kre2-related protein family consisting of Kre2, Ktr1, Ktr2, Ktr3, Ktr4, Ktr5, Ktr6, Ktr7 and Yur1, which share high sequence similarities (Lussier et al., 1999). Kre2, Ktr1 (Romero et al., 1997) and Ktr3 (Lussier et al., 1997) have been shown to possess mannosyltransferase activity in the *O*-linked glycosylation pathway by biochemical and genetic analyses. Yur1 and Ktr2 also appear to have mannosyltransferase activity, as inferred from in vitro measurements of the catalytic activity and a sensitivity assay to K1 killer toxin (Lussier et al., 1996). Ktr6/Mnn6 has been implicated in the transfer of mannosylphosphate to *O*-linked and *N*-linked oligosaccharides (Jigami and Odani, 1999). Golgi localizations of several members of the Kre2 family depend on the function of Vps74, which binds to the short cytoplasmic N-terminal region, and as such, may facilitate the retrograde transport of these proteins to the earlier Golgi compartments or to the ER by the COPI vesicles (Tu et al., 2008; Schmitz et al., 2008). The regions of Kre2 responsible for its traffic or localization were analyzed by switching domains with Pho8 (Lussier et al., 1995) but, as mentioned above, the ER export of Pho8 was later found to be dependent on Svp26 (Bue et al., 2006), which makes the simple interpretation of their results using the chimera proteins difficult. Thus, except for Ktr3, it remained unknown whether the ER exit of Kre2-family proteins are dependent on Svp26 or other adaptor proteins, or distinct mechanisms are operating for their exit from the ER.

Here, we report that the Golgi localization of Kre2 and Ktr1 are dependent on Svp26 as Ktr3 is. Biochemical analysis strongly suggests that Svp26 facilitates the incorporation of Kre2 and Ktr1 into the COPII vesicles. We also show that the Golgi localization of Ktr4, also a member of the Kre2 family, is dependent on the Erv41-Erv46 complex, which was previously identified as a membrane protein complex present abundantly in in vitro generated COPII vesicles (Otte et al., 2001) and immunologically isolated early Golgi compartments (Cho et al., 2000; Cho et al., 2001). The Erv41-Erv46 complex recognizes the lumenal domain of Ktr4 and functions as an adaptor protein for an efficient ER exit of Ktr4.

## RESULTS

### Kre2-family proteins are present at various levels in the cell

**Fig. 1.**
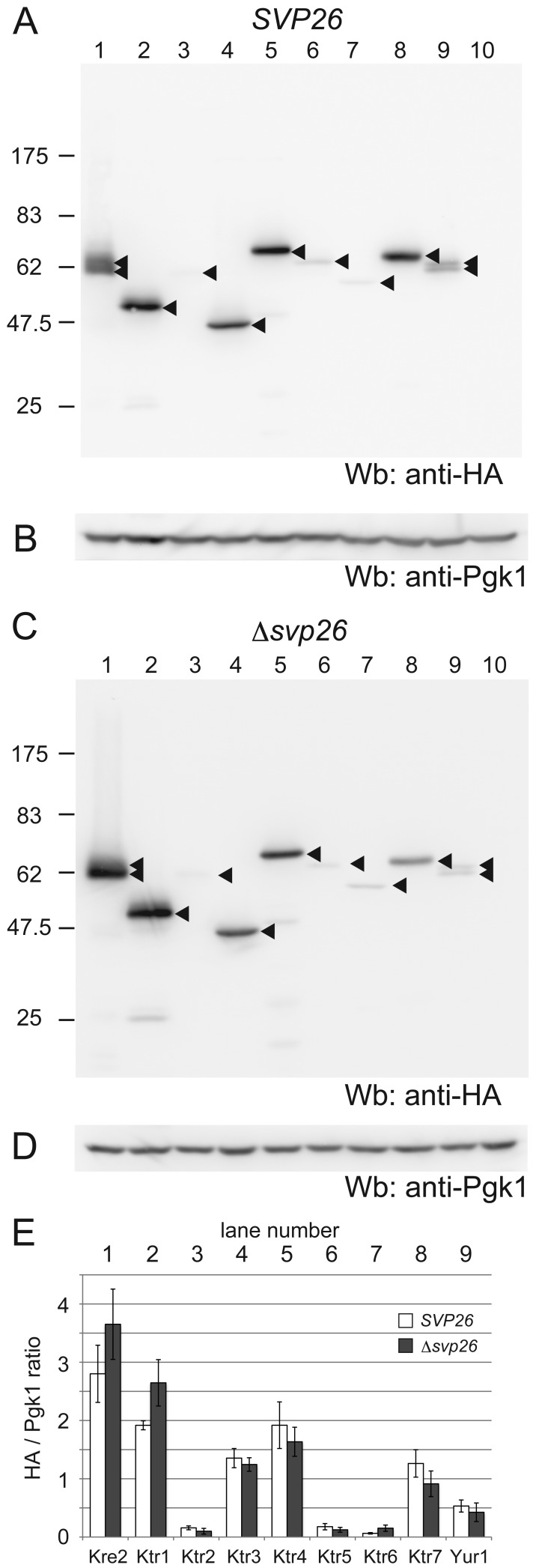
A comparison of expression levels of Kre2-family proteins in wild type and in Δ*svp26* strains. (A,C) C-terminally single HA- (Ktr6) or triple HA- (except for Ktr6) tagged Kre2-family proteins were expressed either in the wild type (A) or in the Δ*svp26* strain (C). Cell lysates were prepared from these strains, separated by SDS-PAGE (10%) and protein levels were compared by immunoblotting with an anti-HA monoclonal antibody. Arrowheads indicate the positions of the HA-tagged Kre2-family proteins. Kre2-3HA and Yur1-3HA appear as doublet bands, which became a single band that ran faster than the doublet by the treatment with EndoH, indicating that the doublets were due to the differences in N-glycosylation. The migration of molecular weight markers is indicated on the left in kilodaltons. The same blots were stripped of anti-HA antibodies and re-probed with an anti-Pgk1 monoclonal antibody (B, wild type and D, Δ*svp26*) as a loading control. Tagged proteins expressed in lanes 1–9 are indicated at the bottom of panel E. The lane 10 contains a cell lysate from a strain in which no protein is tagged (a negative control). (E) Protein amounts for independent experiments (*n* = 3 for wild type, and *n* = 7 for Δ*svp26*) were quantified from immunoblots using Image J and the ratios of signal intensities of anti-HA to those of anti-Pgk1 were graphed. Values represent the mean ± S.D.

To compare the intracellular levels of Kre2-family proteins, isogenic yeast strains of either *SVP26* or Δ*svp26* allele in which each of nine Kre2-family proteins is C-terminally tagged with HA (Ktr6) or 3HA (other Kre2-family proteins) were created (see Materials and Methods). As all members of Kre2-family proteins are predicted to be type II membrane proteins, the HA epitope is expected to face the lumen of the ER or Golgi. Kre2, Ktr1 (Romero et al., 1997) and Ktr3 (Lussier et al., 1997) have been experimentally shown to be involved in the extension of mannose chains in the *O*-linked glycosylation pathway. Ktr6/Mnn6 has been implicated in the transfer of mannosylphosphate to both *O*-linked and *N*-linked oligosaccharides (Jigami and Odani, 1999). Fig. 1 shows Western blots of the Kre2-family proteins (Fig. 1A,C) and the loading control phosphoglycerate kinase (Fig. 1B,D) in equal amounts of lysates of these cells. The amounts of HA-tagged Kre2-family proteins are quantified by scanning the blots (Fig. 1E). The amounts of Kre2-family members are significantly different from each other. Also, the amounts of Kre2, Ktr1 and Ktr6 increased in the absence of Svp26 while the amount of others decreased slightly.

**Fig. 2.**
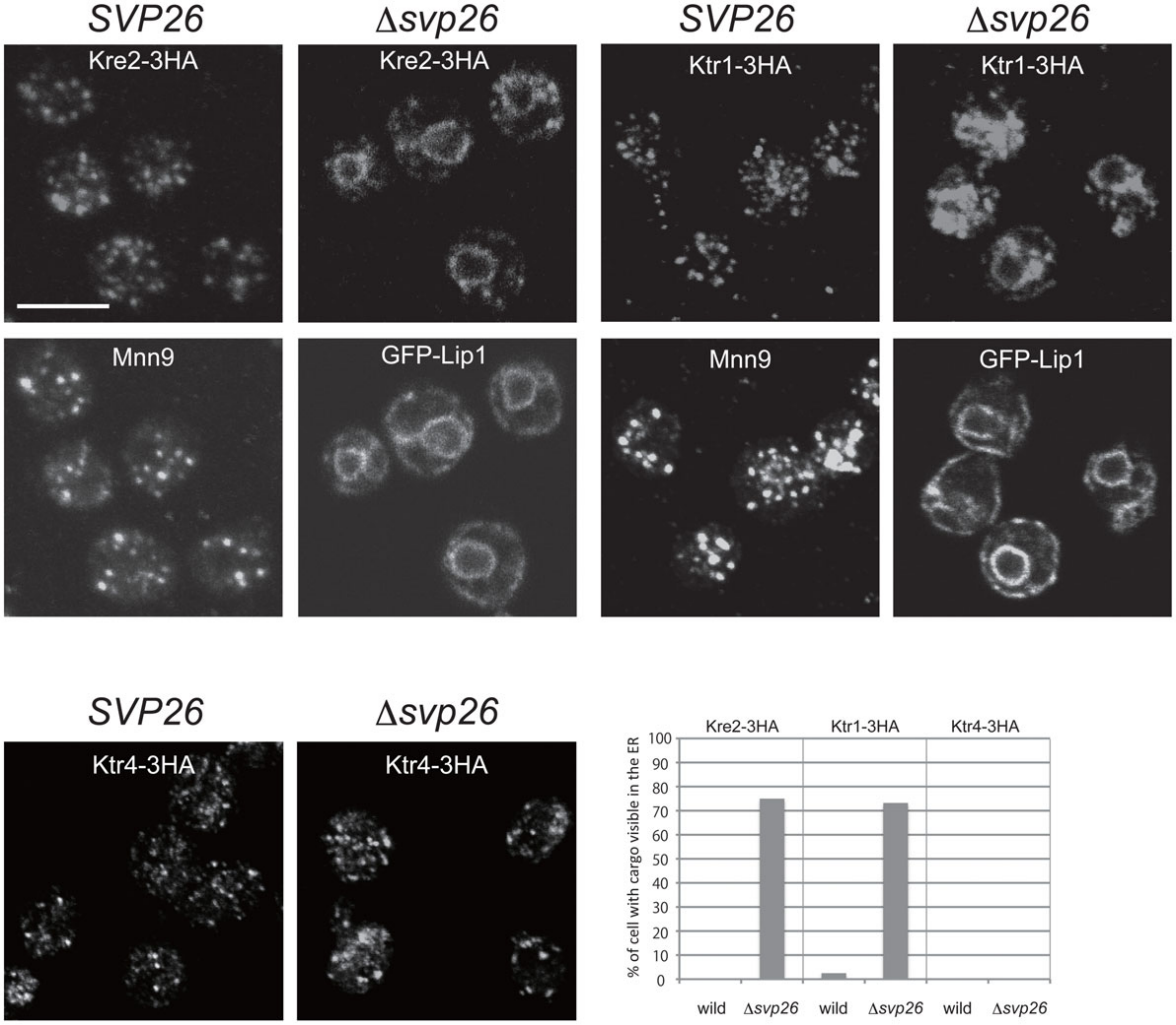
Localization of Kre2, Ktr1 and Ktr4 in the wild-type or Δ*svp26* cells. C-terminally triple HA-tagged Kre2, Ktr1 and Ktr4 were visualized by immunofluorescence staining with mouse anti-HA mAb either in the wild type (left column) or in the Δ*svp26* (right column) strain. The same strains as in Fig. 1 were used. Double-label fluorescence analysis of Kre2-3HA or Ktr1-3HA with Mnn9 or GFP-Lip1 was performed to confirm the localization change of Kre2-3HA and Ktr1-3HA to the ER in the Δ*svp26* strain. Mnn9 and Lip1 are an early Golgi and an ER markers, respectively. Scale bar: 5 µm. The number of cells with cargo clearly visible in the ER were counted and graphed (30≤n≤50).

**Fig. 3.**
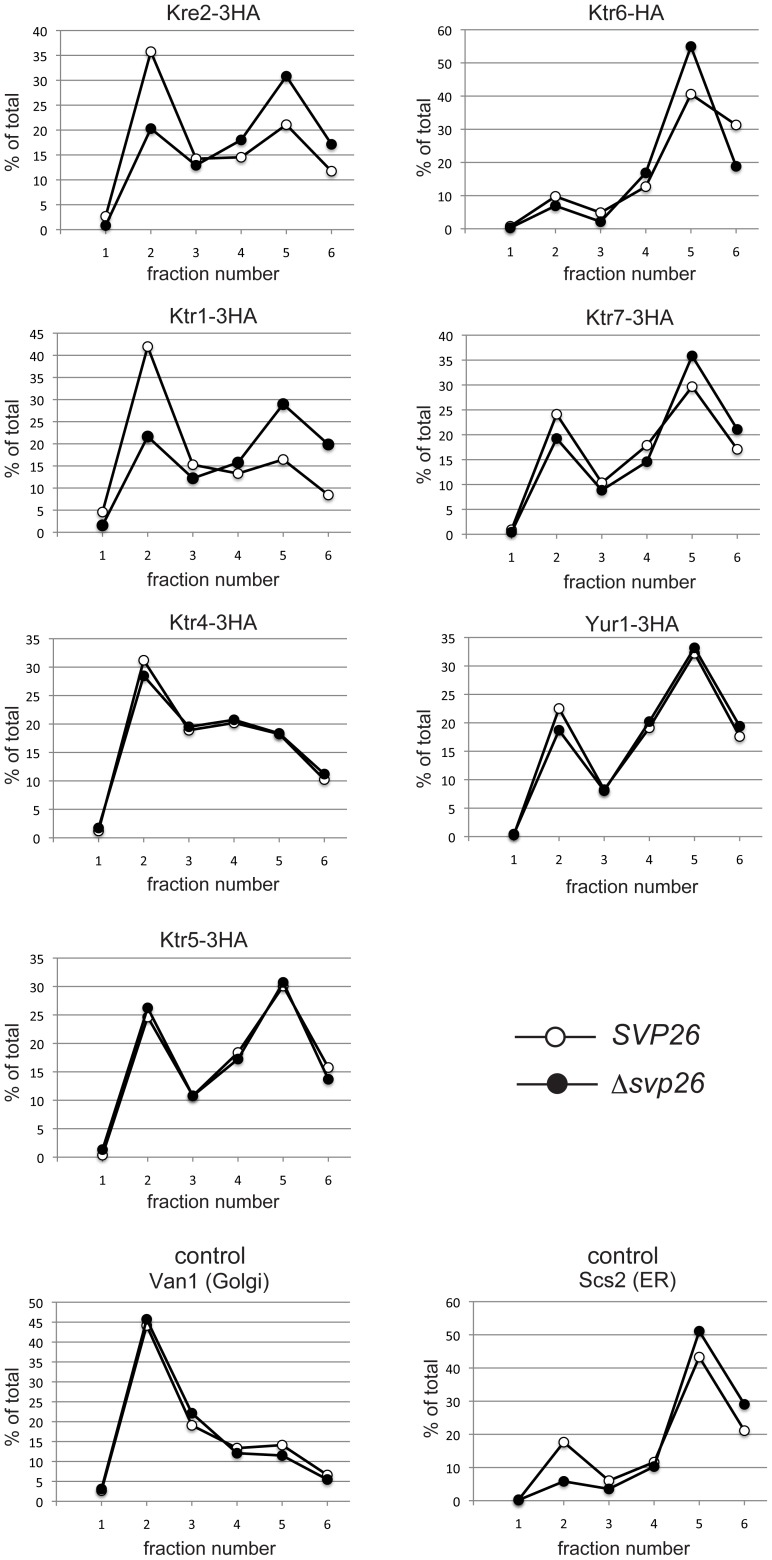
Subcellular fractionation of mannosyltransferases in the wild-type or Δ*svp26* strain. The cell lysate of strains used in Fig. 1 were fractionated on a sucrose density gradient composed of 0.25 ml 60%, 0.5 ml 50%, 1 ml 46%, and 0.25 ml 18% sucrose. After 2.5 h centrifugation in a Beckman TLS55 rotor at 100,000 *g*, 6 fractions of 0.35 ml were sequentially collected from the top of the gradient. Aliquots of each fraction were separated by SDS-PAGE and analyzed by immunoblotting using anti-HA, anti-Van1 (a Golgi marker protein) and anti-Scs2 (an ER marker protein) antibodies. Van1 and Scs2 were detected on the same immunoblot used for detection of Kre2-3HA. The signal intensity of indicated proteins was quantified with Image J software and graphed using Microsoft Excel. The top of the gradient corresponds to fraction number 1 and the bottom corresponds to fraction number 6.

Intracellular localizations of all Kre2-family protein members were then examined by microscopy and cell fractionation, except for Ktr2 and Ktr5, which are present at extremely low levels. Fig. 2 shows the immunofluorescence images of Kre2-3HA, Ktr1-3HA and Ktr4-3HA proteins in the wild-type and Δ*svp26* cells. As signals of Ktr6-HA, Ktr7-3HA and Yur1-3HA were below the detection limit of this study's microscopy, only the results obtained by sucrose density gradient fractionation are shown (Fig. 3). It has already been reported that the Golgi localization of Ktr3 is dependent on Svp26 (Noda and Yoda, 2010). Among the proteins analyzed by indirect immunofluorescence microscopy, localization of Kre2 and Ktr1 changed dramatically from the Golgi (Fig. 2, left column) to the ER (Fig. 2, right column) pattern by deleting the *SVP26* gene. In double-labelling immunofluorescence microscopy, both Kre2 and Ktr1 in the wild-type cells partially overlapped with Mnn9, a subunit of mannosyltransferase complex involved in protein N-glycosylation and a resident of the early Golgi (Hashimoto and Yoda, 1997; Jungmann and Munro, 1998), indicating Kre2 and Ktr1 are localized in different but overlapping Golgi compartments (Fig. 2). The ring stainings of Kre2 and Ktr1 observed in the Δ*svp26* cells coincided with the signals of GFP-Lip1, a known ER-resident protein fused to GFP (Kurita et al., 2011; Vallée and Riezman, 2005) (Fig. 2). Ktr4 were localized to dots indicative of Golgi localization both in the wild-type and Δ*svp26* cells (Fig. 2, bottom two panels). The consistent results were obtained by sucrose density gradient fractionation (Fig. 3). Ktr5, Ktr7 and Yur1 are more abundant in the ER than in the Golgi of both wild-type and Δ*svp26* cells in contrast to Kre2 and Ktr1, and this pattern did not change by deleting the *SVP26* gene. Ktr6 showed a localization pattern of the ER-resident protein in the wild-type cells, which is inconsistent with the fact that Ktr6/Mnn6 is required for the addition of mannosylphosphate to glycoproteins in the Golgi. This distribution was not altered in the Δ*svp26* cells, although deleting the *SVP26* gene resulted in the increased protein level of Ktr6 (Fig. 1E). We are currently investigating the functional relationship between Svp26 and Ktr6. In conclusion, we found that Kre2-family proteins are divided into groups with different intracellular distributions, and in addition to Ktr3 that was reported previously, the Golgi localizations of Kre2 and Ktr1 strongly depend on the function of Svp26. While this study is in progress, Schuldiner and colleagues reported by a large-scale microscopic analysis that Kre2 accumulated in the ER in the absence of Svp26 (Herzig et al., 2012).

### Svp26 is co-immunoprecipitated with some Kre2 family proteins

**Fig. 4.**
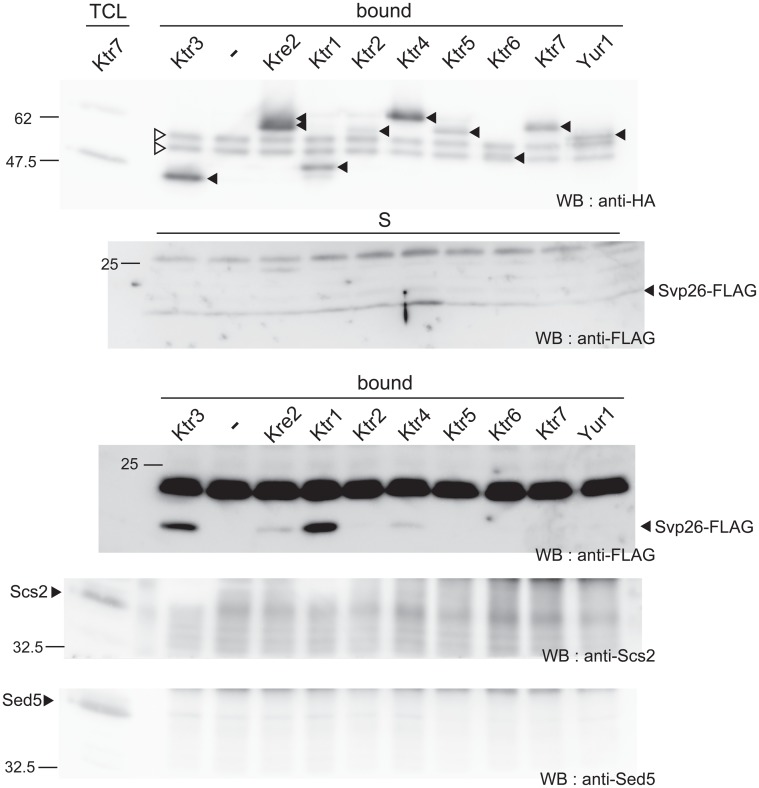
Co-immunoprecipitation experiments of Svp26. The Δ*svp26* strains expressing Kre2 family proteins tagged with an HA epitope at the C-termini (the same proteins used in Fig. 1) were transformed with a *CEN* plasmid expressing the *SVP26-FLAG* construct from a *SVP26* promoter (pHI130). The total cell lysate of these strains were prepared by strong agitation with glass beads and were solubilized with 1% digitonin. After centrifugation, HA-tagged mannosyltransferases were precipitated with anti-HA mAb from the supernatants. Svp26-FLAG in the immunoprecipitates (Bound) and in the input (S) was detected by immunoblotting with an anti-FLAG mAb and indicated by closed arrowheads. Each input lane contains 1% of the total material used for the precipitation. Immunoprecipitated HA-tagged Kre2-family proteins are also detected by Western blotting with anti-HA antibodies (top). Positions of Kre2-family proteins are indicated by closed arrowheads. Immunoglobulin heavy chains are indicated by open arrowheads. A band of Ktr6 overlaps with that of the immunoglobulin heavy chain. The migration of molecular weight markers is indicated at the side of the blots in kilodaltons. Immunoblots of bound fractions with anti-Scs2 and anti-Sed5 are shown as negative controls.

A co-immunoprecipitation experiment was next performed to test if Svp26 would bind to Kre2-family proteins. Δ*svp26* strains expressing HA-tagged Kre2-family proteins, the same strains as used in Fig. 1, were transformed with a *CEN* plasmid expressing Svp26-FLAG from the *SVP26* promoter. Cell extracts were prepared from these strains by agitation with glass-beads and the membrane proteins were solubilized with 1% digitonin. HA-tagged Kre2-family proteins were immunoprecipitated with an anti-HA mAb, and the immunoprecipitates were examined for the presence of Svp26-FLAG using an anti-FLAG mAb. A strain expressing Ktr3-3HA was included as a positive control because the ER exit of Ktr3 is Svp26-dependent and Ktr3 is co-immunoprecipitated with Svp26 very efficiently, as we showed previously (Noda and Yoda, 2010). In this co-immunoprecipitation experiment, the interactions with proteins of low abundance, such as Ktr2, Ktr5, Yur1, may not be detected, especially if the interaction is weak. However, if the co-immunoprecipitation of Svp26 with a particular Kre2-family protein is detected, it indicates the functional relationship between the two proteins. As seen in Fig. 4, the co-immunoprecipitation of Svp26-FLAG with Ktr1-3HA was efficient. In contrast, although its Golgi-localization depends on Svp26, the signal of Kre2-3HA in the immunoprecipitate was very weak, and in some experiments, as weak as the negative control. Ktr4 was also co-immunoprecipitated with Svp26-FLAG very weakly (see also Discussion). The efficient and reproducible co-precipitation would indicate that binding to Svp26 helps Ktr1 to localize in the Golgi. The weak co-immunoprecipitation of Svp26-FLAG with Kre2-3HA also suggests the functional relationship between these proteins.

### Svp26 facilitates the ER exit of Kre2 and Ktr1

**Fig. 5.**
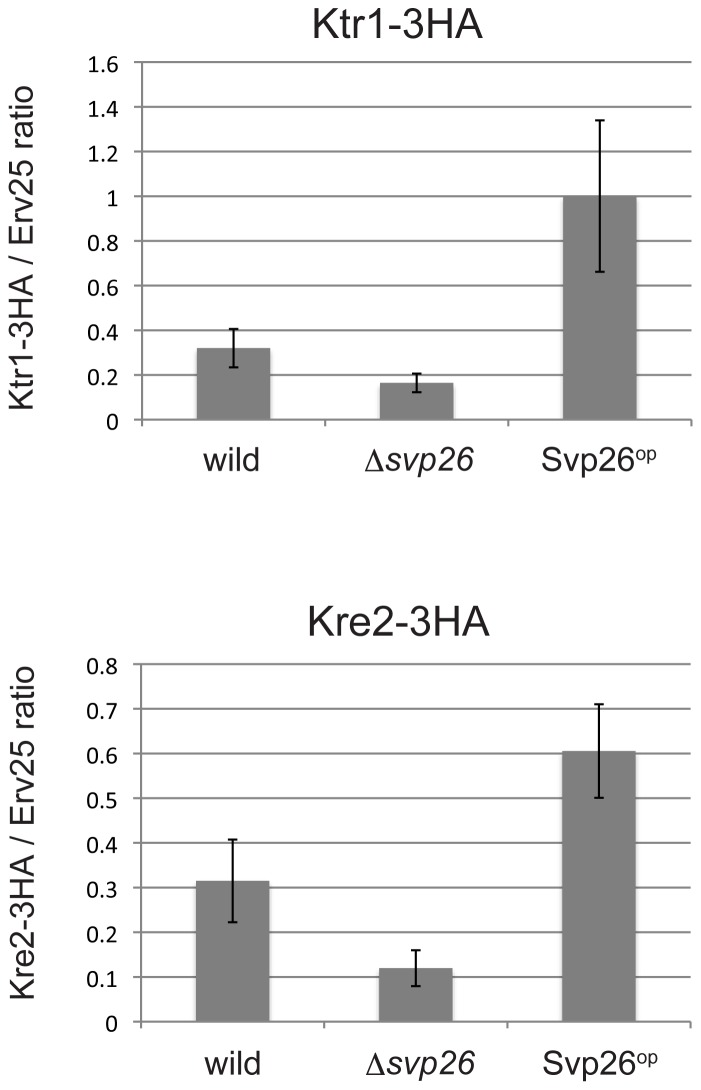
In vitro COPII budding assays using membranes from wild-type, Δ*svp26* or Svp26-overproducing cells. The ER-enriched membrane fractions prepared from the indicated strains were incubated in the presence of purified COPII coat components and the incorporation of Ktr1-3HA (the upper panel) or Kre2-3HA (the lower panel) into COPII vesicles was analyzed by immunoblotting. Incorporation efficiencies of Ktr1-3HA or Kre2-3HA normalized to those of Erv25 are calculated and graphed. Averages from 5 (Ktr1-3HA) and 4 (Kre2-3HA) independent experiments were plotted with standard deviations.

We previously demonstrated that Svp26 functions to facilitate the efficient exit of Ktr3 from the ER (Noda and Yoda, 2010). To test whether Svp26 would also facilitate the ER exit of Kre2 and Ktr1, in vitro COPII vesicle budding reactions were performed. Microsomes prepared from cells expressing Kre2-3HA or Ktr1-3HA from the construct integrated into the chromosomes were incubated with purified COPII subunits, and packaging efficiencies of Kre2-3HA or Ktr1-3HA into COPII vesicles were measured. As shown in Fig. 5, incorporation of Kre2 and Ktr1 into COPII vesicle fractions generated using the microsomal membranes derived from Δ*svp26* cells were significantly lower than when wild-type membranes were used in the reactions. Also, over-production of Svp26 substantially increased the packaging efficiencies of Kre2 and Ktr1 into COPII vesicle fractions. Representative immunoblots are shown in supplementary material Fig. S1. These findings strongly suggest that Svp26 functions as an adaptor protein to facilitate the packaging of Kre2 and Ktr1 into COPII vesicles.

### Ktr4 is mislocalized to the ER in the absence of the *erv41* or *erv46* gene

**Fig. 6.**
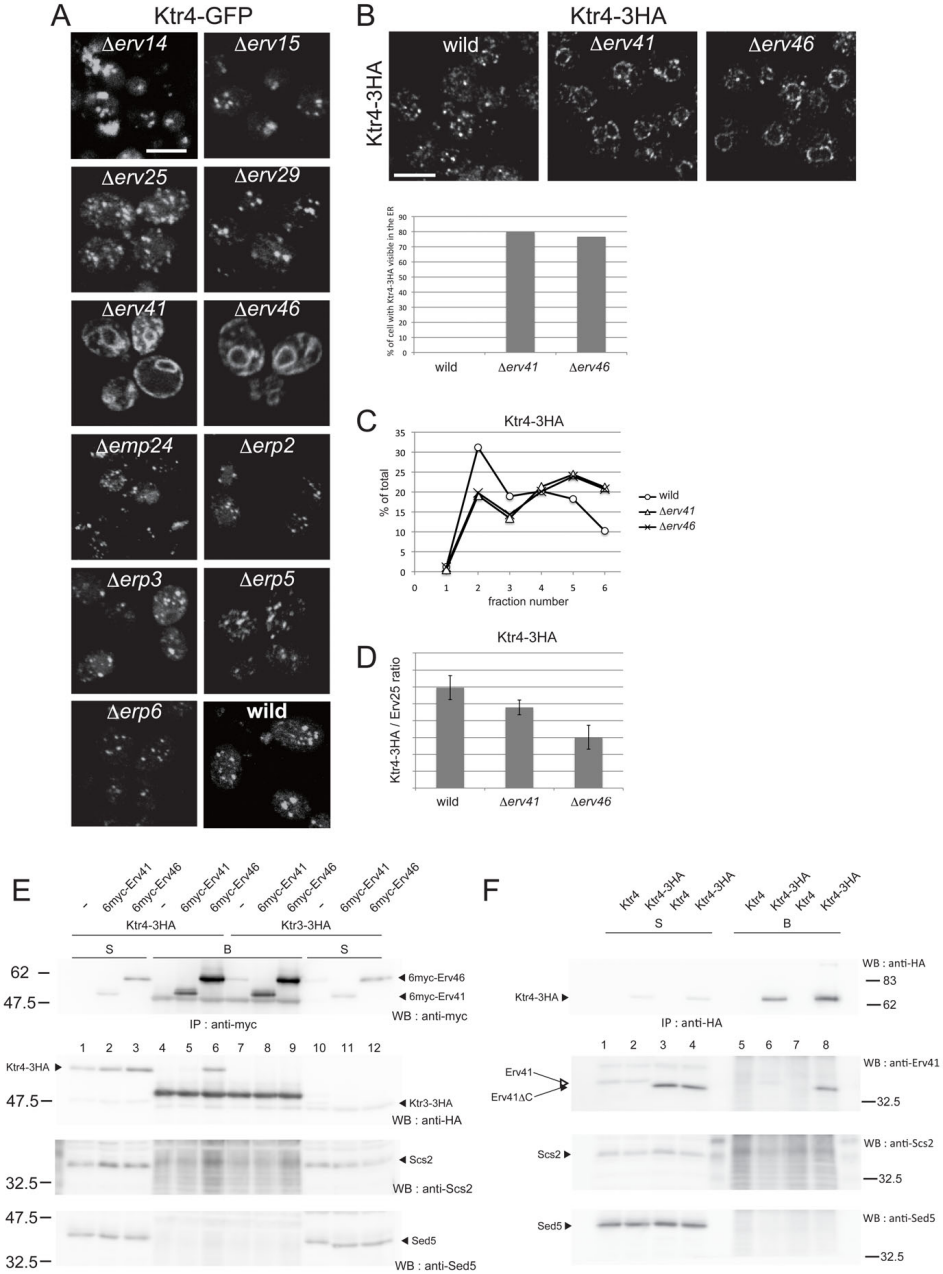
Erv41 and Erv46 are required for Golgi-localization of Ktr4-3HA and for packaging of Ktr4-3HA into COPII vesicles. (A) A screen for genes required for the Golgi-localization of Ktr4-GFP. Disruptants of genes encoding membrane proteins present abundantly in the COPII vesicles and the related proteins were transformed with a *CEN* plasmid expressing Ktr4 fused to GFP at the C-terminus. The fluorescence images of cells expressing Ktr4-GFP in the indicated strains are shown. Scale bar: 5 µm. (B) Immunofluorescence images of Ktr4-3HA in wild-type, Δ*erv41* and Δ*erv46* strains. A chromosomal copy of the *KTR4* gene was replaced with a sequence coding for a C-terminally triple HA-tagged Ktr4 by homologous recombination in the indicated strains. Representative fluorescent images visualized by immunostaining with mouse anti-HA mAb are shown. Scale bar: 5 µm. The number of cells with cargo clearly visible in the ER were counted and graphed (*n* = 30). (C) Subcellular fractionation of Ktr4-3HA in the wild-type, Δ*erv41* or Δ*erv46* strain. Fractionation in a sucrose density gradient was performed as in Fig. 3. (D) Incorporation of Ktr4-3HA into COPII vesicles generated in in vitro reactions using ER-enriched membranes prepared from wild-type, Δ*erv41* or Δ*erv46* strain. Results from 4 independent experiments were plotted as in Fig. 5. (E) Co-immunoprecipitation experiments of Ktr4 with Erv41 and Erv46. The Δ*erv41* or Δ*erv46* disruptants expressing triple HA-tagged Ktr3 or Ktr4 from the chromosome under the control of their original promoters, were transformed with an empty vector (−), or a *CEN* plasmid expressing N-terminally 6× myc-tagged Erv41 or Erv46 under the control of a *YPT1* promoter. Cell lysates were prepared as in Fig. 4 and 6myc-tagged Erv41 or Erv46 was precipitated with anti-myc mAb (9E10). Ktr3-3HA or Ktr4-3HA in the immunoprecipitates (B) and in the input (S) was detected by immunoblotting with anti-HA mAb. Lanes 1–6 contain results from cells expressing Ktr4-3HA, and lanes 7–12 contain those from cells expressing Ktr3-3HA. After the co-immunoprecipitated proteins were detected with anti-HA mAb (the upper panel), the blot was stripped and reprobed with anti-myc mAb to detect the immunoisolated 6myc-Erv41 or 6 myc-Erv46 proteins and with anti-Scs2 and anti-Sed5 antisera as negative controls. (F) Co-immunoprecipitation experiments from the strain producing truncated version of Erv41 (Erv41ΔC). Cell lysates were prepared as in Fig. 4 and the membrane proteins were solubilized with 1% digitonin. Proteins in the input (S) and co-immunoprecipitated proteins with Ktr4-3HA (B) were separated by 10% SDS-PAGE gel and detected by immunoblotting with anti-Erv41, anti-HA, anti-Scs2 (a negative control) and anti-Sed5 (a negative control) antibodies. Strains used in this experiment are described on top of the each lane. The migration of molecular mass markers is indicated on the left in kilodaltons.

Next, gene disruptants were searched for in which Ktr4, one of the Kre2-family members whose Golgi localization does not depend on Svp26, is mislocalized to other organelles. As many of the membrane proteins abundantly present in the COPII vesicles, including Emp and Erv proteins, are reported to function as specific adaptors for the ER exit of secretory and membrane proteins, we examined the localization of Ktr4-GFP expressed from a *CEN*-based plasmid in Δ*erv14*, Δ*erv15*, Δ*erv25*, Δ*erv29*, Δ*erv41*, Δ*erv46*, Δ*emp24*, Δ*erp2*, Δ*erp3*, Δ*erp5* and Δ*erp6* strains. As seen in Fig. 6A, Ktr4-GFP was found in dot-like structures indicative of Golgi localization, in the wild type or in most of the deletion mutants. However, in Δ*erv41* or Δ*erv46* cells, Ktr4-GFP was clearly mislocalized to the ER. We and Barlowe's group independently found and reported that Erv41 and Erv46 form a complex (Otte et al., 2001; Cho et al., 2001). Deletion of one of the genes results in significant reduction or loss of the product encoded by the other gene. Ktr4-3HA produced from the authentic chromosomal allele was also mislocalized to the ER in Δ*erv41* or Δ*erv46* cells (Fig. 6B), and introduction of the *ERV41* or *ERV46* genes on a *CEN* plasmid into these cells restored the Golgi localization of Ktr4-3HA (data not shown). So, the rest of experiments were performed using cells expressing Ktr4-3HA. In good agreement with immunofluorescence data (Fig. 6B), subcellular fractionation in sucrose density gradients showed a peak shift of Ktr4-3HA from a Golgi fraction to an ER fraction in Δ*erv41* or Δ*erv46* cells (Fig. 6C). Furthermore, in vitro COPII budding reactions were performed to examine whether the localization shift of Ktr4-3HA from the Golgi to the ER in the absence of Erv41 or Erv46 occurred as a consequence of a reduction in the incorporation of Ktr4-3HA into the COPII vesicles. Incorporation efficiencies of Ktr4-3HA into COPII vesicle fractions were significantly lower when donor membranes prepared from Δ*erv41* or Δ*erv46* cells were used than when wild-type membranes were used, strongly suggesting that Erv41 and Erv46 facilitate incorporation of Ktr4 into the COPII vesicles probably by functioning as a cargo adaptor protein (Fig. 6D). Representative immunoblots are shown in supplementary material Fig. S1.

### Ktr4 is co-immunoprecipitated with Erv46

We anticipated that, if Erv41 and Erv46 function as an adaptor protein for the ER exit of Ktr4, they might interact with each other. To address this possibility, we performed a co-immunoprecipitation experiment. As Erv41 and Erv46 have sequences necessary for their localization or traffic at the C-terminal cytoplasmic tails (Otte and Barlowe, 2002), 6 myc-tag was appended to their N-terminus and expressed under the control of the *YPT1* promoter on a *CEN* plasmid in Δ*erv41* or Δ*erv46* cells co-expressing Ktr4-3HA or Ktr3-3HA (a negative control) from the chromosomally-integrated construct. Cell extracts were prepared from these strains by agitation with glass-beads and the membrane proteins were solubilized with 1% Triton X-100. Myc-tagged Erv41 or Erv46 was immunoprecipitated with an anti-myc antibody, and the immunoprecipitates were examined for the presence of Ktr4-3HA using an anti-HA monoclonal antibody. Results are shown in Fig. 6E. Ktr4-3HA was co-immunoprecipitated with 6myc-Erv46 efficiently (lane 6). In contrast, only a nonspecific level of Ktr4-3HA was co-immunoprecipitated with 6myc-Erv41 (compare lane 5 with lanes 8 and 9). This was not an anticipated result because it was previously shown that Erv41 and Erv46 form a functional complex. This result will be discussed in the Discussion. Nonetheless, co-immunoprecipitation of Ktr4 with Erv46 indicates that Ktr4 has an ability to bind to at least Erv46 or a complex including Erv46, and via this interaction, Erv46 facilitates the ER exit of Ktr4.

To further analyse involvement of the Erv41-Erv46 complex in ER exit of Ktr4, we created a strain whose *ERV41* gene was genetically manipulated to produce a truncated version of the Erv41 protein lacking its C-terminal 13 amino acids (Erv41ΔC). It was reported that this tail region was required for efficient incorporation of the complex into the COPII vesicles and that the Erv41ΔC-Erv46 complex was accumulated in the ER (Otte and Barlowe, 2002). We performed the sucrose density fractionation analysis and found that the distributions of Erv41ΔC and Erv46 were shifted to the ER-localization pattern as reported. We also found that Ktr4-3HA, but not Van1, was also accumulated more in the ER fractions with concomitant reduction in the Golgi fraction (supplementary material Fig. S2), which is consistent with the idea that the Erv41-Erv46 complex is involved in the ER-Golgi trafficking of Ktr4, probably as an ER exit adaptor. We next performed the co-IP experiment using this strain and found that Erv41ΔC, but not Erv41, was efficiently co-immunoprecipitated with Ktr4-3HA (Fig. 6F). This result probably indicates that the Erv41-Erv46 complex associates with Ktr4 more tightly in the ER than in the Golgi, which is consistent with the regulated binding predicted for adaptor-cargo pairs.

### The lumenal domain of Ktr4 is required for the Erv41 or Erv46-dependent Golgi localization of Ktr4

**Fig. 7.**
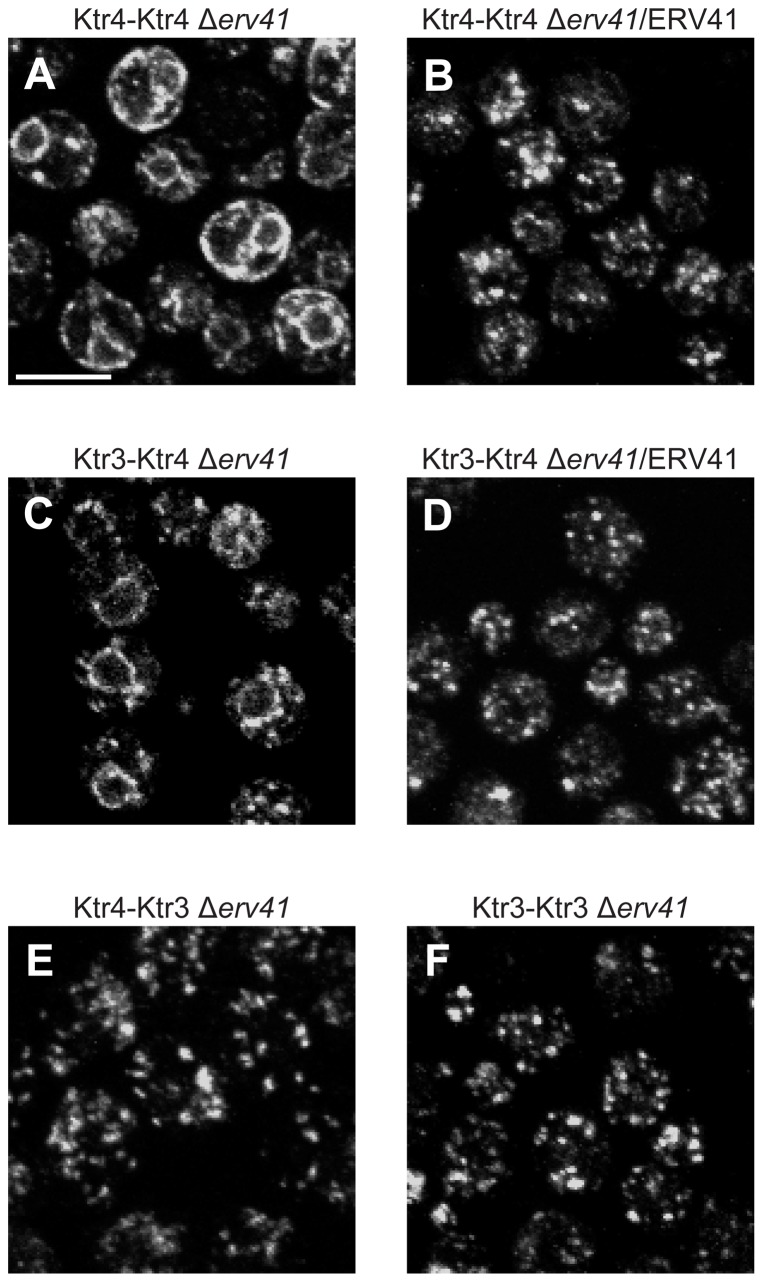
Localization of the Ktr3-Ktr4 and the Ktr4-Ktr3 chimeras in the wild-type and Δ*erv41* cells. The lumenal domains of Ktr3 and Ktr4 were swapped with each other by introducing BglII restriction sites (encoding the amino acids RS) at the junction between the lumenal and the transmembrane domains. A construct Ktr3-Ktr4 indicates a chimera composed of N-terminal cytosolic plus transmembrane domains of Ktr3 and a lumenal domain of Ktr4, and Ktr4-Ktr3 indicates a chimera possessing the opposite domains. Top two panels: localization of the Ktr4-Ktr4 chimera protein. Ktr4-Ktr4 was found in the ER in the Δ*erv41* cells (A). By introducing the *CEN* plasmid carrying *ERV41*, Golgi localization of Ktr4-Ktr4 was restored (B). Middle two panels: localization of the Ktr3-Ktr4 chimera protein. Ktr3-Ktr4, as Ktr4-Ktr4, was found in the ER in the Δ*erv41* cells (C). By introducing the *CEN* plasmid carrying *ERV41*, Golgi localization of Ktr3-Ktr4 was restored (D). Bottom two panels: localization of the chimera proteins possessing a lumenal domain of Ktr3. Both Ktr4-Ktr3 (E) and Ktr3-Ktr3 (F) showed Golgi localization in Δ*erv41* cells. Scale bar: 5 µm.

We previously showed that the lumenal domain of Ktr3 is recognized by Svp26 and required for Svp26-dependent Golgi localization (Noda and Yoda, 2010). To identify the domain of Ktr4 required for the recognition by Erv41 and Erv46, we next examined the localization of chimera proteins between Ktr4, whose Golgi localization is dependent on Erv41 and Erv46, and Ktr3, whose Golgi localization is independent of Erv41 and Erv46. As both Ktr3 and Ktr4 are type II membrane proteins, chimera proteins are created by switching lumenal domains at the position immediately C-terminal to the predicted membrane-spanning region as described in Materials and Methods. A construct Ktr3-Ktr4 indicates a chimera composed of N-terminal cytosolic plus transmembrane domains of Ktr3 and a lumenal domain of Ktr4, and Ktr4-Ktr3 indicates a chimera possessing the opposite domains. Each construct has triple HA-tags at the C-terminus. To facilitate domain switching, a BglII site was generated at the junction between the DNA sequences encoding transmembrane and lumenal domains of Ktr4 by PCR amplification. The BglII site (AGATCT) introduces additional amino acids Arg-Ser at the junction in chimeras. As *KTR3* originally has Arg-Ser codons at this position but the DNA sequence is different from the one recognized by BglII, it was changed to AGATCT by PCR. The immunofluorescence images of the Δ*erv41* cells expressing these chimera proteins are shown in Fig. 7. The Ktr4 protein possessing this additional Arg-Ser (Ktr4-Ktr4) in the Δ*erv41* cells was localized to the ER (Fig. 7A), and the Golgi localization was restored by the introduction of the *ERV41* gene on the *CEN* plasmid (Fig. 7B), indicating that the introduction of Arg-Ser at this position did not interfere with the normal trafficking of Ktr4. The protein levels of the chimeras were quantified by immunoblotting with anti-HA and normalized to the level of Pgk1. When the mean value of Ktr4/Pgk1 in Δ*erv41* strain was set to 1, Ktr3-4/Pgk1 in Δ*erv41* was 1.16±0.12 (mean, S.D.) and Ktr4-4/Pgk1 Δ*erv41* was 1.61±0.31. Ktr3-Ktr4 was found localized to the ER in the Δ*erv41* cells (Fig. 7C), and the introduction of the *ERV41* gene restored the Golgi localization of this chimera protein (Fig. 7D). In contrast, Ktr4-Ktr3 and Ktr3-Ktr3 exhibited the clear Golgi localizations in Δ*erv41* cells (Fig. 7E,F). Localization of these chimera proteins in Δ*erv46* cells showed basically the same patterns as in Δ*erv41* cells (data not shown). Thus, the lumenal domain of Ktr4 is required for its Erv41- and Erv46-dependent Golgi localization.

## DISCUSSION

Svp26 recognizes the lumenal domain of the Ktr3, which is a type II membrane protein, and facilitates its exit from the ER (Noda and Yoda, 2010). We attempted to determine the domains of Kre2 and Ktr1 necessary for their Svp26-dependent Golgi localization. As both Kre2 and Ktr1 are type II membrane proteins (Lussier et al., 1995), chimera proteins with Ktr4 were expressed in Δ*svp26* cells and their localizations were examined. Ktr4-Ktr1, a chimera protein composed of the cytoplasmic C-terminal domain plus the transmembrane domain of Ktr4 and the lumenal domain of the Ktr1 exhibited the ER localization in the Δ*svp26* cells, and the chimera construct possessing the opposite domains (Ktr1-Ktr4) exhibited the Golgi localization in the Δ*svp26* cells (supplementary material Fig. S3). These results strongly suggest that, similarly to Ktr3, the Svp26 recognizes the lumenal domain of Ktr1 and facilitate its ER export. In contrast, the Ktr4-Kre2 chimera unexpectedly exhibited the ER localization even in the wild-type cells (supplementary material Fig. S3). As domains are switched at the junction between the transmembrane and the lumenal domains in these chimera proteins, this result may indicate that both the transmembrane and the lumenal domains, or the entire Kre2 protein is required for recognition by Svp26.

As shown in the current study, Erv41 and Erv46 recognize the lumenal domain of Ktr4. One of the biological significances of binding to the lumenal domain of the mannosyltransferases would be that adaptors may be inhibiting the enzymatic activity of the mannosyltransferases by associating with the catalytic domain during their exit from the ER. We previously found that Svp26 binds to the lumenal domain of Mnn2, an α-1, 2-mannosyltransferase that extends mannose branches in *N*-linked glycans, and facilitate the ER exit of Mnn2 (Noda and Yoda, 2010). It was also shown that in Δ*svp26* cells, secretory proteins receive hyper *N*-glycosylation (Inadome et al., 2005). Mnn2 may exhibit enzymatic activity in the earlier stages of the secretory pathway in the absence of Svp26 than in the wild-type cells, which may lead to the hyper *N*-mannosylation of the secretory proteins in the Δ*svp26* cells. Pho8 and Gda1, other cargoes of Svp26, are also type II membrane proteins and the catalytic sites probably reside at their lumenal domains. It has been reported that, as Ktr3 and Mnn2, the lumenal domain of Pho8 is also recognized by Svp26 (Bue et al., 2006). We are currently attempting to narrow the region involved in the association with the adaptors by creating more finely-designed chimera proteins. Testing the involvement of the DXD motif, which is considered to constitute the catalytic site of the glycosyltransferases (Wiggins and Munro, 1998), in association with the adaptors, may be interesting. Alternatively, the association in the lumen of the organelles can be regulated by a simple mechanism such as pH changes upon moving between the organelles.

From its definition, an adaptor facilitates the cargoes to exit from the ER through protein-protein interaction. As Kre2 and Ktr1 similarly depend on Svp26 to localize in the Golgi, it is anticipated that binding between Svp26 and Kre2 or Ktr1 would be detected by co-immunoprecipitation experiments. However, as shown in Fig. 4, while Svp26 was reproducibly co-immunoprecipitated with Ktr1, it was co-immunoprecipitated with Kre2 only very weakly. This may be explained in several ways. Firstly, Kre2 may be more labile in the detergent extracts than Ktr1 and tends to be released from Svp26. Secondly, as an adaptor-cargo interaction occurs transiently in the ER and should be lost in the Golgi, proteins transported rapidly from the ER to the Golgi may have less chance of being associated with the ER exit adaptor proteins. Also, Ktr4, whose Golgi-localization appears to be independent of Svp26 was co-immunoprecipitated, albeit very weakly, with Svp26. Although this may be due to a non-specific interaction between the membrane proteins, it is also speculated that some cargoes may be dependent on more than one adaptor for their exit from the ER in varying degrees. Kre2 or Ktr1 residing on the Golgi in the absence of Svp26 (Fig. 3) may be transported out of the ER with the aid of such additional adaptor proteins besides Svp26.

The recent study by Herzig et al., that showed the wide-range search for the cargo-adaptor pairs using the technology they developed and named PAIRS, found no cargo proteins whose localization was significantly affected in the Δ*erv41* cells among the 375 candidate cargo proteins they tested (Herzig et al., 2012). Therefore, the Erv41-Erv46 complex appears to function specifically as an ER exit adaptor protein for Ktr4 in yeast. We also observed the localization of Mnn2-GFP in Δ*erv41* and Δ*erv46* cells, and found that Mnn2-GFP localized to the Golgi in these deletion mutants as in the wild-type cells (supplementary material Fig. S4). Erv41 and Erv46 are conserved proteins and have putative orthologues in *Caenorhabditis elegans*, *Drosophila melanogaster*, and *Homo sapiens*. A mammalian homologue of Erv46 (mErv46) and the yeast Erv41-Erv46 complex were shown to be localized to the ER and early Golgi and recycle between these organelles, yet their precise molecular functions have been unknown (Orci et al., 2003; Otte et al., 2001; Breuza et al., 2004). In this study, we show that the Erv41-Erv46 complex functions as an adaptor to facilitate ER exit of Ktr4. This is the first demonstration of the molecular function of this conserved protein complex. The homologues of Erv41 and Erv46 in other species may also function to facilitate ER exit of the mannosyltransferases.

Ktr4 shares 36% similarity in an amino acid sequence with Kre2 (Lussier et al., 1999), and as can be seen from Fig. 1, is abundantly present in the cell. In the yeast genome database (Saccharomyces Genome Database), it is indicated that deletion of the *KTR4* gene displayed a synthetic negative interaction with deletion of the *ANP1* gene, which encodes a subunit of the mannosyltransferase complex that extends α-1,6-linked mannoses in *N*-glycans, and that the Δ*ktr4* strain showed an abnormality in bud morphology. From these data, it is speculated that Ktr4 possibly functions as a mannosyltransferase in an *N*-linked or *O*-linked glycosylation pathway, though neither the in vivo nor in vitro enzymatic activity of Ktr4 has been reported as far as we searched the literature. In the database, it is also indicated that the deletion of the *ERV41* or *ERV46* gene displayed a synthetic growth defect with deletion of the *ANP1* gene, which may suggest that the correct Golgi localization of Ktr4 is important for its physiological function.

In the experiments in Fig. 6E, Ktr4-3HA was efficiently co-immunoprecipitated with 6myc-Erv46, but co-immunoprecipitation with 6myc-Erv41 was not detected. This was an unanticipated result as Erv41 and Erv46 forms a functional complex. The complex formation between Erv41 and Erv46 was confirmed by using anti-Erv41 and anti-Erv46 antibodies in the experiments in Fig. 6E. However, it was noticed that the protein level of 6myc-Erv46, which was expressed from the *YPT1* promoter on the *CEN* plasmid, was much higher than that of the endogenous Erv46 protein (supplementary material Fig. S5). The level of 6myc-Erv41 was approximately the same as the endogenous Erv41. From these results, it was speculated that excess 6myc-Erv46 that failed to associate with Erv41 and stayed in the ER was co-immunoprecipitated with Ktr4-3HA. It is currently being tested whether Erv41 or Erv46 would bind to Ktr4 stronger in the ER than in the later organelles such as the Golgi, and how these interactions are regulated.

A large number of proteins in eukaryotic cells are transported into the lumen of the ER. And then, they either stay in the ER without ever leaving the ER, leave the ER to the early Golgi and travel from there to the later organelles, or return from the Golgi to the ER via the retrograde transport pathway. However, the precise mechanisms by which they are sorted into these distinct pathways remain unclear. Efforts towards comprehensively identifying the ER exit adaptor proteins and their cargo proteins, and elucidating their traffic will further advance our understanding of the mechanisms that regulate the ER-Golgi traffic.

## MATERIALS AND METHODS

### Strains, plasmids, media, and reagents

*S. cerevisiae* strains used in this study are listed in Table 1. All strains, except for gene disruptants used in Fig. 6A, which were purchased from EUROSCARF, are derivatives of KA31a, with genes disrupted or epitope tagged by homologous recombination. All DNA fragments generated by PCR amplification for plasmid construction were checked by DNA sequencing.

**Table 1.**
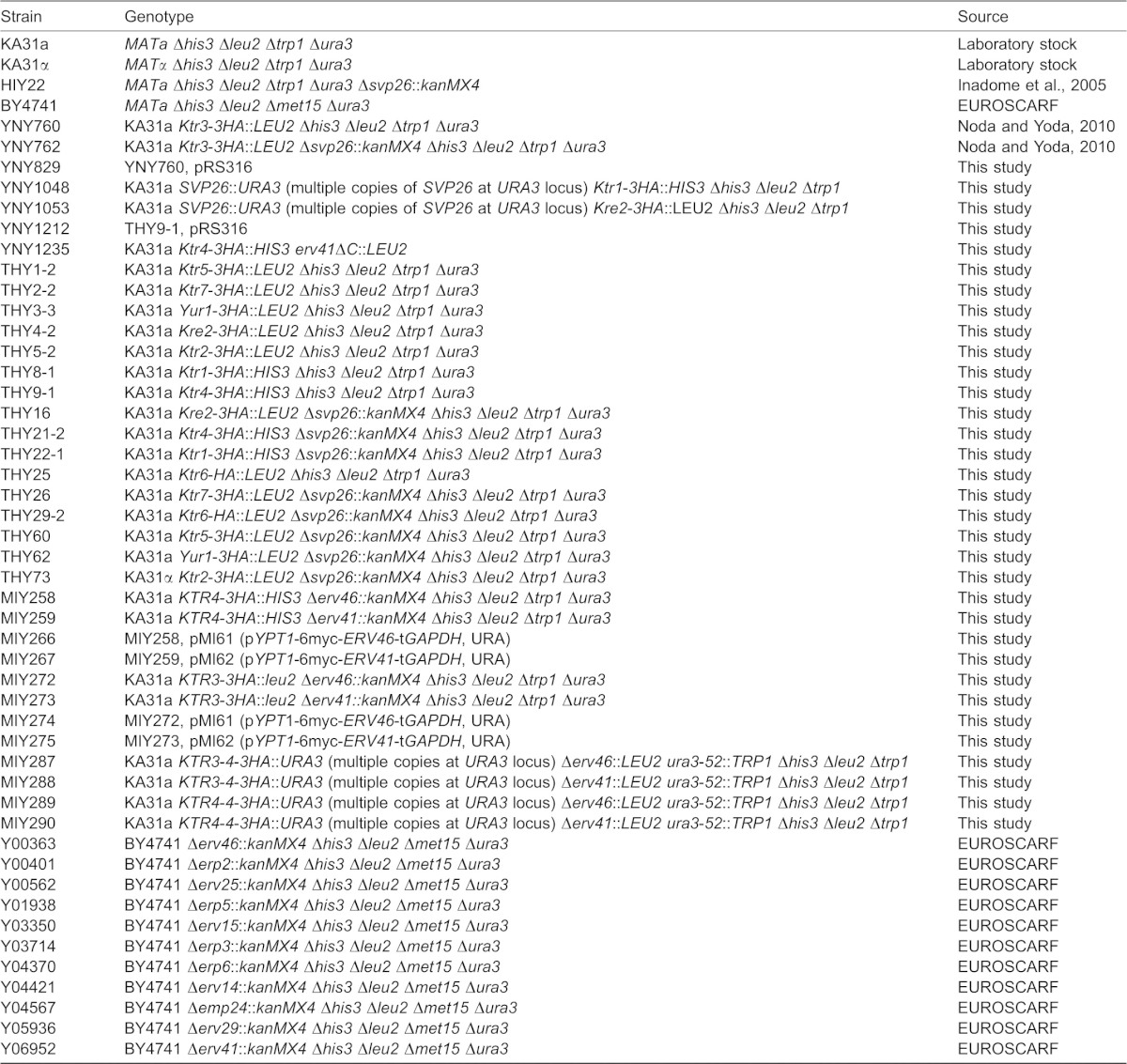
*S. cerevisiae* strains used in this study

For tagging Kre2-family proteins with three copies of the HA epitope at their C-termini, an appropriate DNA fragment of the 3′-region of each protein was amplified by PCR and cloned in pYN497 (*HIS3* marker) or pYN503 (*LEU2* marker) as described previously (Noda and Yoda, 2010). These plasmids carry a coding sequence for triple HA followed by a *TDH3* terminator. The sequences of primers used are available upon request. These plasmids were linearized by cutting at a unique restriction site located within a cloned region of each ORF, and were transformed into appropriate yeasts to obtain strains with chromosomally tagged genes by homologous recombination.

For the overexpression of *SVP26* (Fig. 5), a DNA fragment including an ORF of *SVP26* and the franking regions were amplified by PCR, and cloned into BamHI/SalI-digested pRS306. The resulting plasmid, pYN574, was linearized by digestion at an StuI site in a *URA3* gene in the vector plasmid, and was transformed into a yeast strain carrying an *ura3-52* allele. Among the *URA3* colonies, cells overproducing the Svp26 protein at appropriate levels due to multiple integration of the plasmid at the *ura3-52* locus were selected by immunoblotting of the lysates with an anti-Svp26 antiserum. The resulting strain, YNY938, was crossed to other strains, sporulated and dissected to yield strains used in Fig. 5.

To create a *URA3 CEN* plasmid expressing *KTR4*-*GFP*, a DNA fragment containing a 5′-promoter region and the ORF of the *KTR4* was amplified by PCR, placing an SpeI site upstream of the start codon and an XhoI site immediately upstream of a stop codon. The resulting product was digested with SpeI and XhoI, and ligated into the similarly digested expression vector, pCA115, yielding pMI34. To create a plasmid expressing N-terminally 6myc-tagged Erv46, the ORF region was amplified by PCR, placing a BamHI site immediately upstream of the start codon and an SalI site downstream of the stop codon. The resulting fragments were digested with BamHI and SalI, and ligated into the similarly digested expression vector, pYN345, yielding pMI61, in which the *YPT1* promoter was placed upstream of the *6MYC*-*ERV46* sequence, and the *TDH3* terminator was placed downstream of it. A plasmid expressing N-terminally 6myc-tagged Erv41 (pMI62) was created in an identical manner, except that an additional step to remove the intron by PCR was necessary.

A strain that produces C-terminally 13 amino acids truncated version of Erv41, YNY1235, was created by homologous recombination using the appropriate plasmid in a manner similar to C-terminally HA-tagging of the Kre2-family proteins.

Strains expressing chimeras that were used in Fig. 7 were created in a similar manner as previously described (Noda and Yoda, 2010). The colonies with the expression levels of chimera constructs comparable to the wild-type genes were selected by Western blotting.

Yeast cells were grown in YPD [1% Bacto yeast extract (BD Biosciences, Franklin Lakes, NJ), 2% Bacto peptone (BD Biosciences), and 2% glucose] or SD [0.17% yeast nitrogen base without amino acids (BD Biosciences), 0.5% ammonium sulphate, 2% glucose, and appropriate supplements] medium at 30°C. *Escherichia coli* DH5α (F^−^, *supE44 ΔlacU169 φ80lacZDM15 hsdR17 recA1 endA1 gyrA96 thi-1 relA1*) was used in plasmid propagation. *E. coli* was grown in an LB [1% Bacto tryptone (BD Biosciences), 0.5% Bacto yeast extract (BD Biosciences) and 0.5% NaCl] medium. Digitonin was purchased from Wako Pure Chemical Industries (Japan).

### Antibodies, immunoblotting and indirect immunofluorescence

Antisera against Scs2, or Erv41 and Erv46 were gifts from Dr Satoshi Kagiwada (Nara Women's University, Nara, Japan) and Dr Charles Barlowe (Dartmouth medical school, USA), respectively. Anti-Van1 and anti-Mnn9 antisera were produced in our laboratory as previously described (Hashimoto and Yoda, 1997). Anti-myc (9E10, Berkeley Antibody), anti-HA (12CA5, Roche Diagnostics, Indianapolis, IN) and anti-FLAG (M2, Sigma-Aldrich) monoclonal antibodies were purchased. For immunoblotting, anti-HA, anti-Van1 or anti-Scs2 antiserum was used at a dilution of 1/200, 1/1000 or 1/2000, respectively. The intracellular localizations of HA-tagged proteins were observed by indirect immunofluorescence as previously described (Noda and Yoda, 2010). Anti-HA mouse monoclonal antibody was used as a primary antibody. Alexa 488-conjugated goat antibody to mouse immunoglobulin G (Molecular Probes) was used as a secondary antibody. YNY1214 and YNY1215 were used for a double-labelling experiment with an ER marker, GFP-Lip1 (Fig. 2).

### Sucrose density gradient fractionation

Subcellular fractionation in a sucrose density gradient was performed as described previously (Noda and Yoda, 2010). Briefly, cell lysates were prepared by suspending yeast spheroplasts by 10 strokes in a Dounce homogenizer in an ice-cold 1× JR lysis buffer (20 mM HEPES, 50 mM potassium acetate, 0.2 M sorbitol, 2 mM EDTA, pH 7.4) (Shimoni and Schekman, 2002) containing protease inhibitors (1 µg/ml each of chymostatin, aprotinin, leupeptin, pepstatin A, antipain, 1 mM benzamidine and 1 mM phenylmethylsulfonyl fluoride), and unlysed cells were removed by a centrifugation at 400 *g* for 3 min. An aliquot of the supernatant was diluted in a 2% SDS solution and absorbance at 280 nm was measured. After diluting the supernatant to A^280^ = 0.35, 0.2 ml was loaded onto a sucrose step gradient, which was generated using the following steps [all sucrose solutions were made (w/v, %) in 20 mM HEPES-KOH, pH 7.4, 50 mM potassium acetate, 2 mM EDTA]: 0.25 ml 60%, 0.5 ml 50%, 1 ml 46%, and 0.25 ml 18% sucrose. After 2.5 h of centrifugation in a Beckman TLS55 rotor at 100,000 *g*, 6 fractions of 0.35 ml were sequentially collected from the top of the gradient. Aliquots of each fraction were mixed with an SDS sample buffer, and proteins were resolved by SDS-PAGE and detected by immunoblotting using appropriate antibodies. Since the abundance of Ktr6 in each fraction is not high enough for detection by Western blotting, glycosylated proteins including Ktr6-HA were concentrated from each fraction by mixing with ConA-Sepharose beads for overnight in the presence of 0.2% Triton X-100 prior to Western blotting. Enhanced chemiluminescence signals were captured by an image analyzer equipped with a cooled charge-coupled-device camera (LAS-1000plus; Fuji Film, Tokyo, Japan), and digital images were quantified using Image J software and graphed in Microsoft Excel.

### Immunoprecipitation

Co-immunoprecipitation experiments were performed as previously described (Noda and Yoda, 2010).

### In vitro COPII vesicle budding assay

Purification of COPII coat components Sar1, Sec23/24 and Sec13/31, and the vesicle budding assay were performed as previously described (Shimoni and Schekman, 2002; Noda and Yoda, 2010). In Fig. 5, microsomal membranes were prepared from THY8-1 (*KTR1-3HA*::*HIS3 SVP26*), THY22-1 (*KTR1-3HA*::*HIS3* Δ*svp26*), YNY1048 (*KTR1-3HA*::*HIS3 SVP26^OP^*), THY4-2 (*KRE2-3HA*::*LEU2 SVP26*), THY16 (*KRE2-3HA*::*LEU2* Δ*svp26*) and YNY1053 (*KRE2-3HA*::*LEU2 SVP26^OP^*), and used as donor membranes in the budding reactions. In Fig. 6D, microsomal membranes form THY9-1 (*KTR4-3HA*::*HIS3 SVP26*), MIY259 (*KTR4-3HA*::*HIS3* Δ*erv41*) and MIY258 (*KTR4-3HA*::*HIS3* Δ*erv46*) were used for the budding reactions. After the reaction, samples were separated by SDS-PAGE followed by immunoblotting with anti-HA, and packaging efficiency was quantified from chemiluminescence signals in a total membrane fraction and in an MSS fraction, in which COPII vesicles generated in a reaction were concentrated.

## Supplementary Material

Supplementary Material
