# Supplementary material for: Distinct adaptor proteins assist exit of Kre2-family proteins from the yeast ER
Source: Biol Open. 2014 Feb 28;3(3):209–24. doi: 10.1242/bio.20146312 (PMC4001239; doi:10.1242/bio.20146312)
Supplement: Supplementary Material [file supp_3_3_209__index.html]

Distinct adaptor proteins assist exit of Kre2-family proteins from the yeast ER — Distinct adaptor proteins assist exit of Kre2-family proteins from the yeast ER — Supplementary Material 

# Distinct adaptor proteins assist exit of Kre2-family proteins from the yeast ER

## bio.20146312 Supplementary Material

**Files in this Data Supplement:**

- Supplementary Material - Yoichi Noda et al. doi: 10.1242/bio.20146312
