# Supplementary material for: Distinct adaptor proteins assist exit of Kre2-family proteins from the yeast ER
Source: Biol Open. 2014 Feb 28;3(3):209–24. doi: 10.1242/bio.20146312 (PMC4001239; doi:10.1242/bio.20146312)
Supplement: Supplementary Material [file supp_bio.20146312_bio.20146312-s1.pdf]

Biology Open (2014) 000, 1–12 doi:10.1242/bio.20146312

Supplementary Material
Yoichi Noda et al. doi: 10.1242/bio.20146312

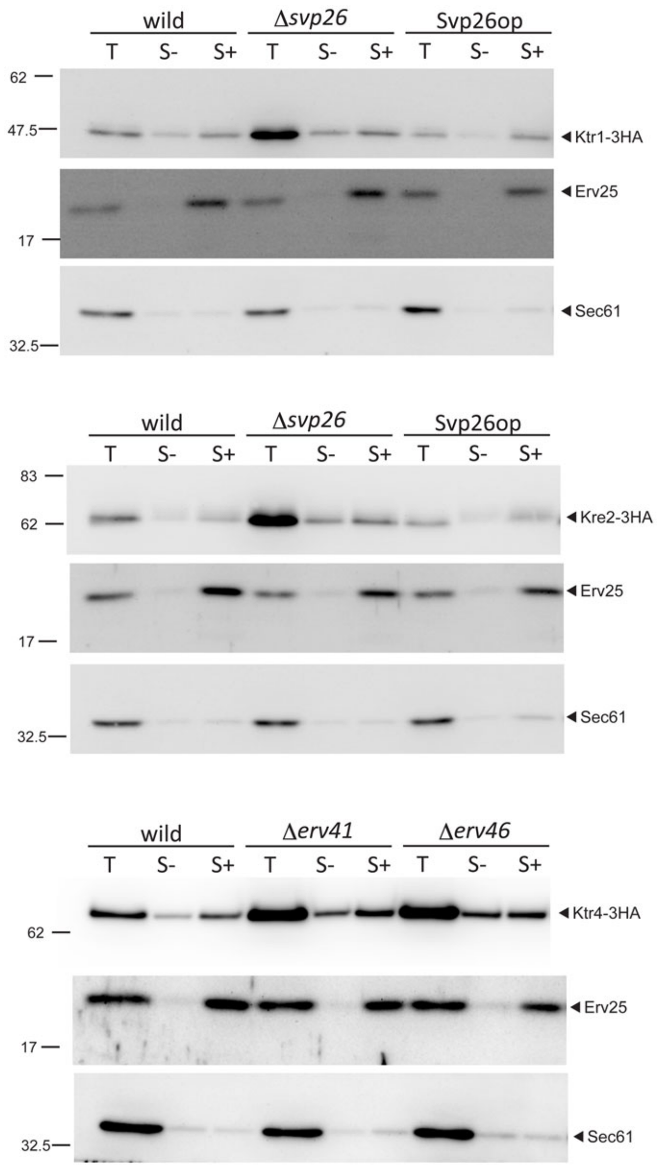

Fig. S1. Immunoblots of COPII budding experiments. Representative immunoblots used to calculate the incorporation efficiency of Ktr1, Kre2 and Ktr4 into the COPII vesicles. The ER-enriched membrane fractions prepared from the indicated strains were incubated either in the presence (+) or absence (−) of purified COPII coat components and the incorporation of Ktr1, Kre2 or Ktr4 into COPII vesicles was analyzed by immunoblotting. T contains 2.5% of the total reaction mixture and S contains 75% of the total COPII-vesicle fraction, both of which were collected after the reaction. Erv25 and Sec61 were monitored as a positive and negative control of the experiment, respectively.

Biology Open (2014) 000, 1–12 doi:10.1242/bio.20146312

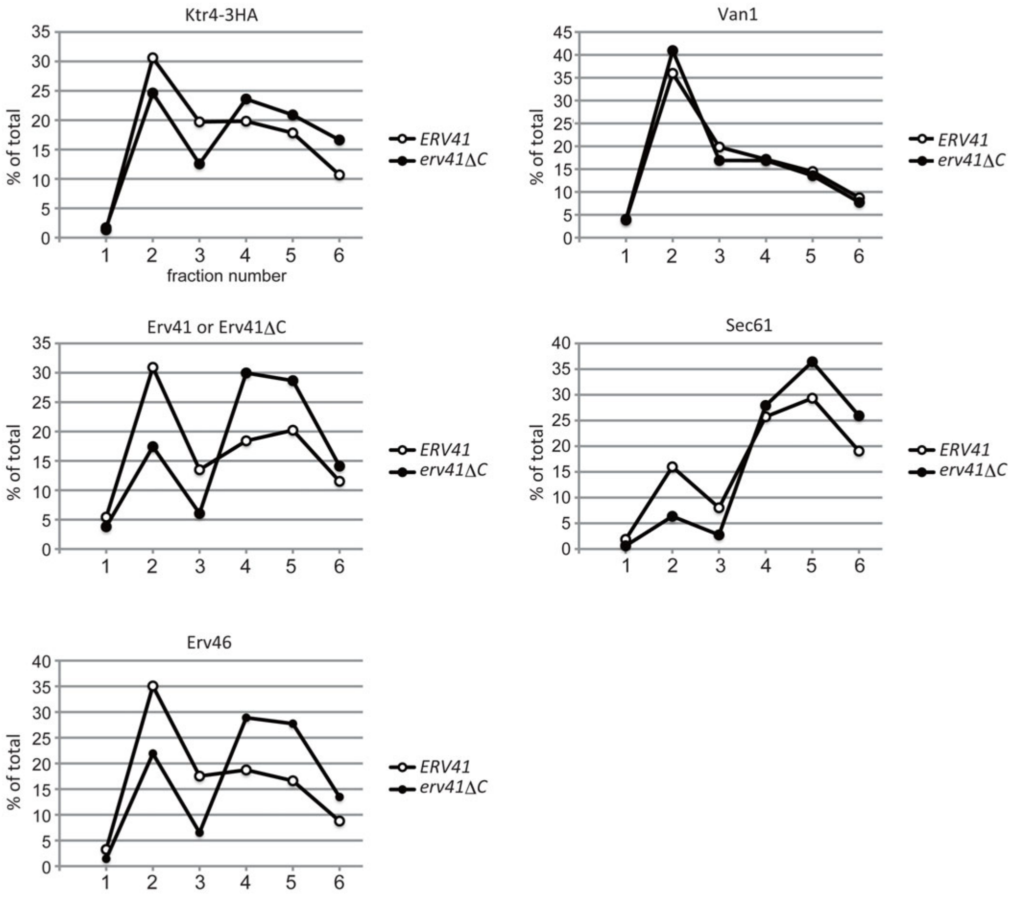

Fig. S2. Subcellular fractionation of Ktr4-3HA, Erv41, Erv41ΔC and Erv46 in the wild-type or erv41ΔC strain. The cell lysate of wild type or *erv41ΔC* were fractionated on a sucrose density gradient composed of 0.25 ml 60%, 0.5 ml 50%, 1 ml 46%, and 0.25 ml 18% sucrose. After 2.5 h centrifugation in a Beckman TLS55 rotor at 100,000 g, 6 fractions of 0.35 ml were sequentially collected from the top of the gradient. Aliquots of each fraction were separated by SDS-PAGE and analyzed by immunoblotting using anti-HA, anti-Erv41, anti-Erv46, anti-Van1 (a Golgi marker protein) and anti-Scs2 (an ER marker protein) antibodies. The signal intensity of indicated proteins was quantified with Image J software and graphed using Microsoft Excel. The top of the gradient corresponds to fraction number 1 and the bottom corresponds to fraction number 6.

Biology Open (2014) 000, 1–12 doi:10.1242/bio.20146312

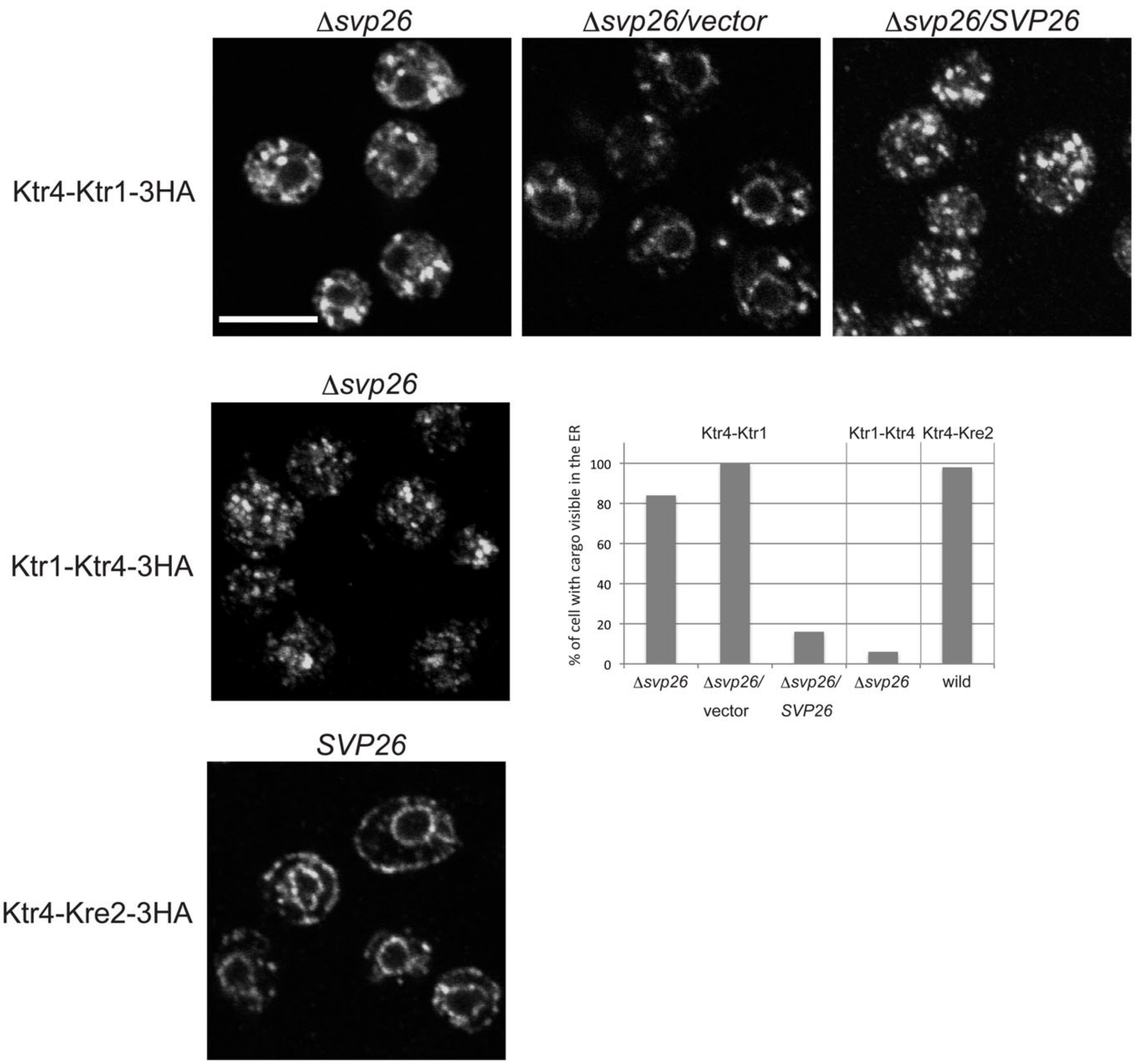

Fig. S3. Localization of the Ktr4-Ktr1, Ktr1-Ktr4 and Ktr4-Kre2 chimeras in the wild-type and Δ*svp26* cells. The lumenal domains of Ktr4 and Ktr1, and Ktr4 and Kre2, were swapped with each other by introducing BglII restriction sites (encoding the amino acids RS) at the junction between the lumenal and the transmembrane domains. A construct Ktr4-Ktr1 indicates a chimera composed of N-terminal cytosolic plus transmembrane domains of Ktr4 and a lumenal domain of Ktr1, and Ktr1-Ktr4 indicates a chimera possessing the opposite domains. Top panels: localization of the Ktr4-Ktr1 chimera protein. Ktr4-Ktr1 was found in the ER in the Δ*svp26* cells (left). By introducing the *CEN* plasmid carrying *SVP26*, Golgi localization of Ktr4-Ktr1 was restored (right). Middle panel: Ktr1-Ktr4 was found in the Golgi in the Δ*svp26* cells. Bottom panel: Ktr4-Kre2 was found in the ER in wild-type cells. Scale bar: 5 µm. The number of cells with cargo clearly visible in the ER were counted and graphed ($n=50$).

Biology Open

Biology Open (2014) 000, 1–12 doi:10.1242/bio.20146312

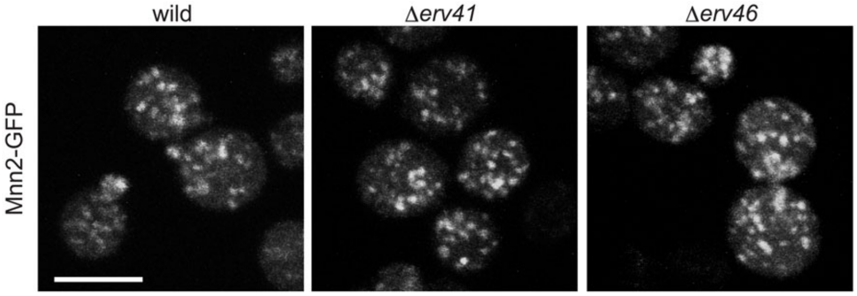

Fig. S4. Localization of Mnn2-GFP in the wild-type, Δ*erv41* or Δ*erv46* cells. A plasmid expressing C-terminally GFP-tagged Mnn2 were introduced into the indicated strains and visualized by fluorescence microscopy. Scale bar: 5 μm.

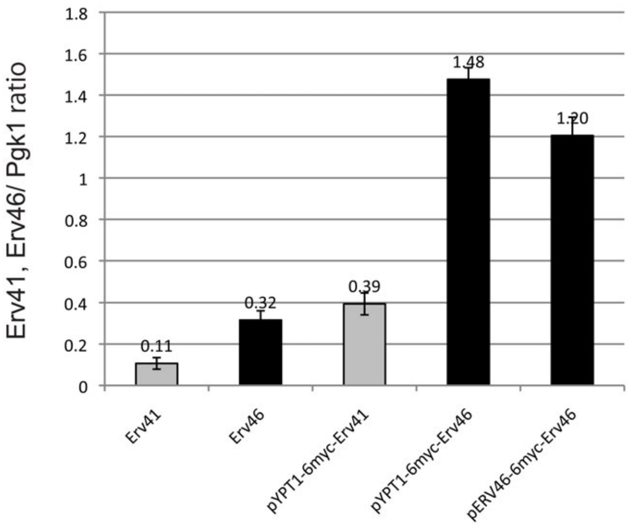

Fig. S5. Comparison of protein levels of endogenous or myc-tagged Erv41 and Erv46. Protein extracts were prepared either from the wild type, or strains producing 6myc-tagged Erv41 or Erv46 by boiling the collected cells in the SDS-PAGE sample buffer. Endogenous and myc-tagged Erv41 and Erv46 were quantified by immunoblotting with anti-Erv41 and anti-Erv46 antisera and normalized to the protein levels of Pgk1 that were detected and quantified with anti-Pgk1 antibodies.

Biology Open
